# A Modified Device for Pressurized Planar Electrochromatography and Preliminary Results with On-Line Sample Application

**DOI:** 10.1007/s10337-013-2430-x

**Published:** 2013-03-05

**Authors:** Aneta Hałka-Grysińska, Piotr Ślązak, Andrzej Torbicz, Mieczysław Sajewicz, Tadeusz H. Dzido

**Affiliations:** 1 Department of Physical Chemistry, Medical University of Lublin, Chodźki 4a, 20-093 Lublin, Poland; 2 Institute of Chemistry, Silesian University, Szkolna 9, 40-006 Katowice, Poland

**Keywords:** Pressurized planar electrochromatography (PPEC), PPEC device, On-line sample application/injection

## Abstract

Pressurized planar electrochromatography (PPEC) is a separating technique in which an electric field is applied to force the mobile phase movement through a porous media (electroosmotic effect). High separation efficiency, fast separations and changes in separation selectivity in comparison to liquid chromatography, especially thin layer chromatography (planar chromatography, TLC), are features of this technique. Constructional methodological challenges to PPEC are obstacles to its development and application in laboratory practice. In this article, an attempt to overcome the challenges related to device construction and sample application/injection is described. The introduced device enables both prewetting of the adsorbent layer and electrochromatogram development with a single PPEC device. It also enables simultaneous application/injection of six samples on a chromatographic plate in a stream of the mobile phase (on-line application/injection). In addition, the PPEC chamber was equipped with a thermostat. The device is characterized by an impressive throughput in comparison to the other planar technique, TLC/HPTLC. Although the developed device still needs improvement, it is, in our opinion, a considerable step toward possible automation of this planar separation technique.

## Introduction

Pressurized planar electrochromatography was first introduced by Nurok et al. [1] in 2004. Since then, a number of articles have been published showing the advantages of this relatively new technique compared with conventional thin-layer chromatography, including high efficiency, the short time needed for the separation process and different separation selectivities [1–14]. Despite these advantages, the potential of this method is not currently used in routine analysis, inter alia due to difficulties with the two stages of the PPEC process that precede the development of electrochromatograms: application of samples on the chromatographic plate and prewetting of an adsorbent layer of the chromatographic plate with a mobile phase solution. Our group has designed pre-wetting reservoirs with partitions, which protected the substance zone applied on the adsorbent layer against elution and dispersion during the prewetting process [6, 10, 11, 13]. Disadvantages of this approach are the risk of losing some of the sample and the need for too many manual operations [13]. The last device proposed by our group was equipped with two partitions in the PPEC chamber, which enabled prewetting of the adsorbent layer with samples zones on it and the separation process in a single unit [14]. However, before running the separation process with this device, a prewetting solution of the mobile phase had to be removed from the PPEC chamber to enable the cover to press the adsorbent layer of the chromatographic plate. In our opinion, this modification has provided an important development stage for the PPEC. In this article, the improved version of the device with one partition instead of two (as previously reported [14]) is described. In the new device, the stages of plate transfer from the prewetting container to a separation device and removal of the mobile phase solution from the PPEC chamber were eliminated, so the risk of losing some of the sample was minimized, and the number of manual operations needed was reduced.

In this article, we also show a new mode of on-line sample application on the chromatographic plate during the PPEC process in the prototype device, which enables running the PPEC separation with six different samples simultaneously. In this the sample application of the technique, pre-wetting of the adsorbent layer and the separation process were performed in an individual device, contrary to what has been reported previously.

## Experimental

### Chemicals and Materials

The chromatographic glass plates RP-18W and LiChrospher RP-18W F_254_ were supplied by Merck (Darmstadt, Germany). Chemicals and solvents were of analytical reagent grade. Methanol and acetonitrile were purchased from POCh (Gliwice, Poland). Citric acid monohydrate was supplied by Merck (Darmstadt, Germany) and disodium hydrogen phosphate by Standard (Lublin, Poland). Bidistilled water was obtained in the laboratory. The components of silicone sealant solutions Sarsil W, Sarsil H50 and hardener (Utwardzacz W) were purchased from Zakłady Chemiczne Silikony Polskie (Nowa Sarzyna, Poland). Pabianickie Zakłady Farmaceutyczne Polfa S.A (Pabianice, Poland) kindly supplied standards of acetaminophen, propyphenazone, caffeine and cefalgin preparations. Acetanilide was from POCh (Gliwice, Poland).

Buffer solution was prepared by mixing a 0.1 M solution of citric acid and 0.2 M solution of disodium hydrogen phosphate in the appropriate ratio and then diluting it with bidistilled water.

### Preparation of Standard Solutions and Samples

In experiments for the calibration curve preparation, various quantities of the standards of caffeine, propyphenazone and acetaminophen in the ranges 0.006–0.04, 0.001–0.086 and 0.02–0.17 g were accurately weighed and dissolved in 50 mL of methanol, respectively, and additionally in 100 mL of methanol for the lowest concentrations of these standards. The number of calibration points was equal to 7.

In the procedure for precision determination, 20 tablets of cefalgin (each containing 150 mg of propyphenazone, 50 mg of caffeine and 250 mg of acetaminophen), with an average weight of 0.7022 g, were finely powdered, and the appropriate amount of powder (about 0.2 g) was accurately weighed and transferred to six 50-mL volumetric flasks containing about 25.0 mL of methanol. The suspension was shaken for 30 min, then diluted to the volume with methanol and filtered using Whatman no. 21 filter paper. The clear solution was applied to the chromatographic plate using the semiautomatic applicator Linomat 5 (Camag, Muttenz, Switzerland) equipped with a 100-mL microsyringe. In order to determine the accuracy, approximately 0.1 g of the tablet mass and 0.006, 0.007 or 0.0086 g of caffeine, 0.0165, 0.024 or 0.026 g of propyphenazone, and 0.0276, 0.036 or 0.044 g of acetaminophen were accurately weighed into nine 50-mL volumetric flasks containing about 25 mL of methanol. The next steps were performed according to the procedure described above.

For preparation of the calibration curve using the internal standard addition method, 0.05–0.1 g of acetaminophen and about 0.06 g of acetanilide (internal standard) were accurately weighed and dissolved in 10 mL of methanol. The number of calibration points was equal to 6. In the procedure for precision determination, 20 tablets of paracetamol (each containing 500 mg of acetaminophen) were weighed, and their average weight was calculated (0.7952 g). The tablets were finely powdered, and the appropriate amounts of powder (about 0.25 g) and internal standard (about 0.15 g) were accurately weighed and transferred to six 25-mL volumetric flasks containing about 15.0 mL of methanol. The suspension was shaken for 30 min, then diluted to the volume with methanol and filtered using Whatman no. 21 filter paper. The clear solution was injected into the PPEC separation system using the on-line homemade application/injection device. In order to determine accuracy, approximately 0.05 g of the tablet mass, 0.06 g of acetanilide and 0.015, 0.03 or 0.043 g of acetaminophen were accurately weighed to nine 10-mL volumetric flasks containing about 5 mL of methanol. The next steps were performed according to the procedure described above.

### PPEC Experiments

#### Chromatographic Plate Preparation

Chromatographic plates were cut into 10 × 5-cm pieces, washed with methanol for 1 min and after drying in air were activated in an oven for 15 min. Then, 4-mm-wide margins were produced with sealant solution on the whole periphery of the adsorbent layer of each chromatographic plate [12].

**Fig. 1:**
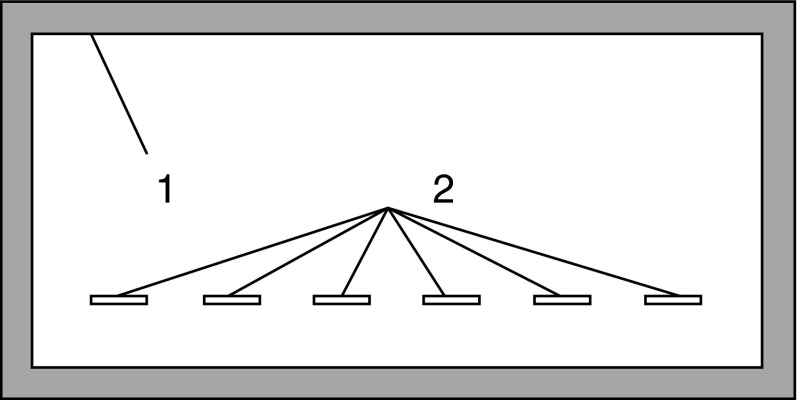
Schematic drawing of the chromatographic plate (5 × 10 cm) with the sealant margin (*1*) on the adsorbent layer periphery and special grooves (*2*) for sample solutions (narrow channels, 1 mm wide and 7 mm long, scratched on the adsorbent layer)

**Fig. 2:**
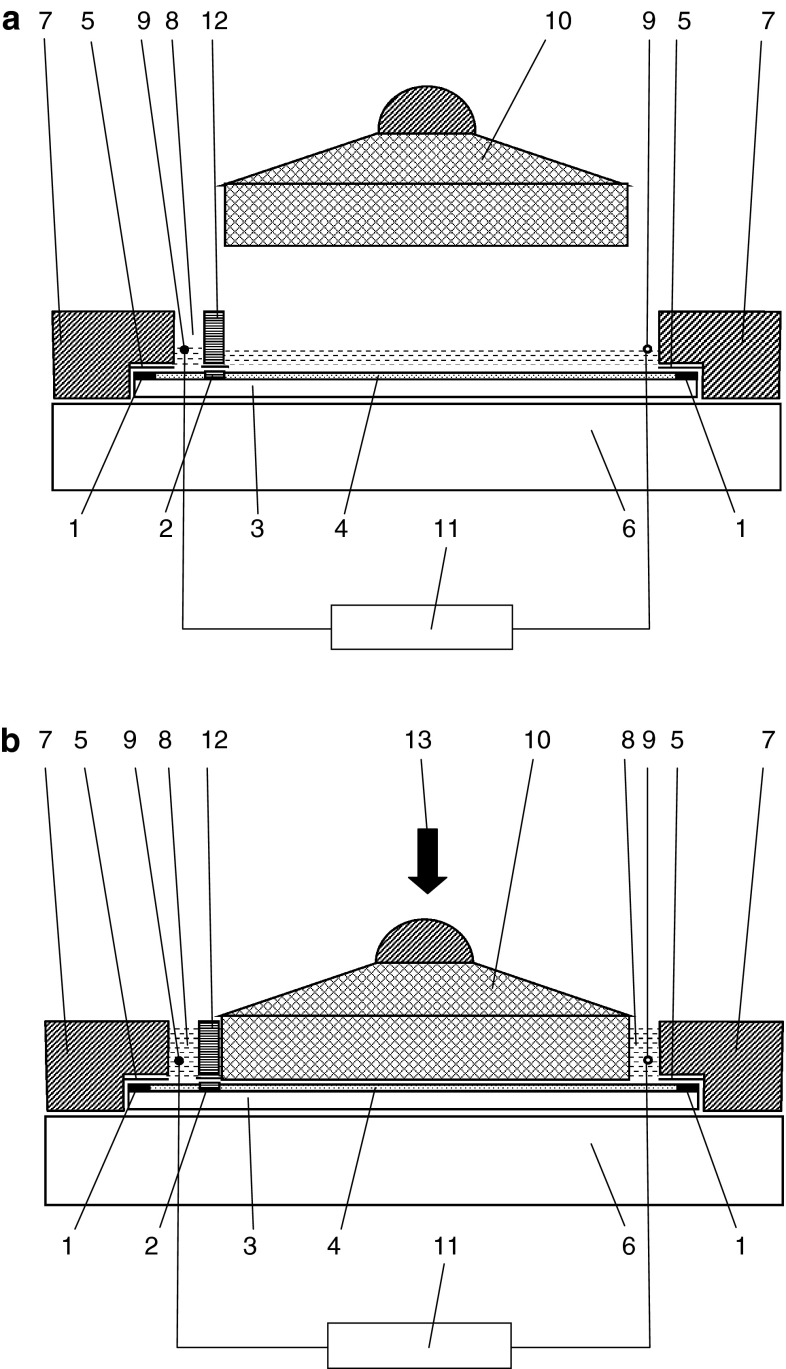
**a** Schematic view of the PPEC chamber during prewetting of an adsorbent layer; **b** schematic view of the PPEC chamber during the separation process. *1* Sealant margin on the adsorbent layer, *2* substance zone or groove in the case of on-line sample application/injection, *3* chromatographic plate, *4* adsorbent layer, *5* Teflon foil, *6* base, *7* frame of the chamber, *8* electrode compartment with the mobile phase solution, *9* electrodes, *10* cover, *11* high voltage power supply, *12* partition, *13* external hydraulic press; patent pending

**Fig. 3:**
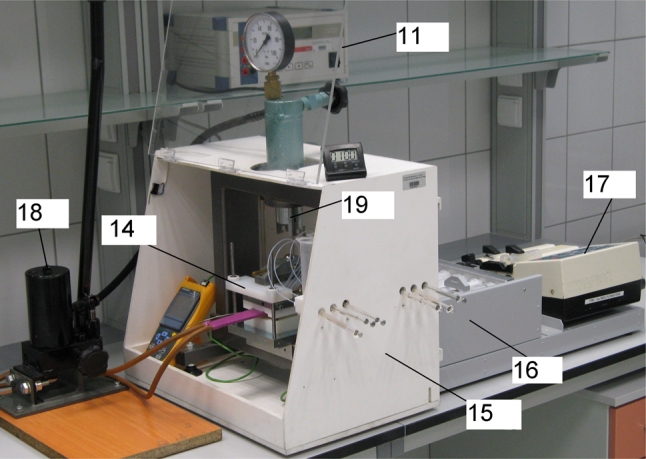
Picture of the PPEC device with on-line sample application/injection; *11* high-voltage supply, *14* PPEC chamber, *15* cabinet for the PPEC chamber, *16* syringe holder, *17* stepping motor, *18* hydraulic pump, *19* hydraulic press

**Fig. 4:**
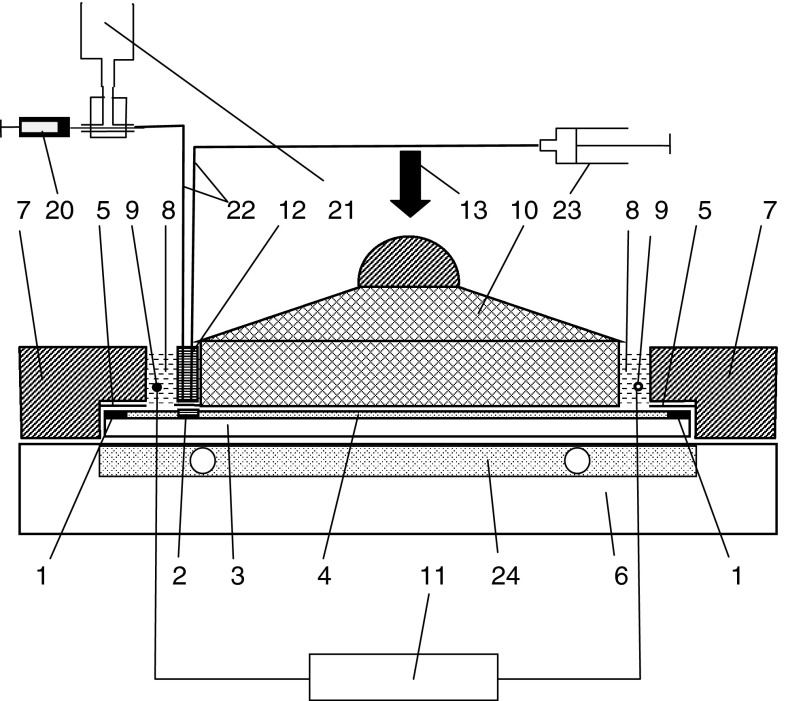
Conceptual view of the PPEC device with on-line sample application; *1* sealant margin on the adsorbent layer, *2* substance zone or groove in the case of on-line sample application, *3* chromatographic plate, *4* adsorbent layer, *5* Teflon foil, *6* base, *7* frame of the chamber, *8* electrode compartment with the mobile phase, *9* electrodes, *10* cover, *11* high voltage power supply, *12* partition, *13* external hydraulic press, *20* microsyringe, *21* reservoir, *22* Teflon tube, *23* syringe, *24* cooling plate; patent pending

When the PPEC equipment with the on-line application/injection device was used, chromatographic plates required additional preparation: on the adsorbent layer, special grooves (2) were produced (narrow channels, 1 mm wide and 7 mm long, were scratched on the adsorbent layer) for sample solutions (Fig. 1). The grooves enabled the sample solution to flow through it from the Teflon tubing inlet to the Teflon tubing outlet (see Figs. 2, 3, 4 and the next sections).

#### Equipment for PPEC

In the first variant, PPEC experiments were performed with the device composed of the PPEC chamber (Fig. 2a, b), high-voltage power supply, EV 262 (Consort, Turnhout, Belgium), hydraulic press (Współpraca, Lublin, Poland) and thermocouple TM-711 Xs (Tenmars, Taipei, Taiwan). In Fig. 2a, b, the PPEC device is equipped with one partition (12), which facilitates prewetting of the adsorbent layer with sample zones (2) applied to it. When the cover was moved down to press the adsorbent layer, the solution was automatically transferred to the cathode compartment (8); see Fig. 2b.

In the second variant, the PPEC device was additionally equipped with an on-line application/injection system and cooling plate combined with the thermostat; see Figs. 3 and 4.

#### Procedure of Sample Application and Electrochromatogram Development

When the PPEC device was used with an aerosol mode of sample application, the solutions (10 μL) of solutes were applied on the plates along its 10-cm edge as 10-mm-long bands by means of the Linomat 5 semiautomatic applicator (Camag, Muttenz, Switzerland). Then the chromatographic plate (3) was placed (with the adsorbent layer face up) in the PPEC chamber, and then the frame (7) of the PPEC chamber and the glass partition (12) were pressed to the chromatographic plate [so that the starting solute zones (2) and sealant margins (1) on the plate were tightly covered with the partition (12) and the frame (7), respectively (Fig. 2a)]. On the subsequent stage, the adsorbent layer (4) was prewetted with the mobile phase solution for 1 min; see Fig. 2a. After prewetting, the cover (10) of the PPEC chamber was pressured against the adsorbent layer using the hydraulic press (13), and then the potential was applied to the electrodes and the separation process started (Fig. 2b). The maximum potential separation distance was approximately equal to the length of the cover (10), i.e. 30 mm. After the separation process, when the potential was switched off, the plate was removed from the PPEC device and dried in air for 30 min, and the chromatograms were registered with the TIDAS TLC 2010 Scanner (J&M, Aalen, Germany).

When the device was used with on-line sample application/injection (Figs. 3, 4), the chromatographic plate with special grooves (2) in the adsorbent layer (4) was placed in the chamber, then the frame (7) of the PPEC chamber and the glass partition (12) equipped with six lines of Teflon tubing for sample solutions (in Fig. 4 a conceptual view of one line of Teflon tubing is shown) were pressed on the chromatographic plate (3). The grooves (2) in the adsorbent layer (4) and the sealant margins (1) on the plate were tightly covered with the partition (12) and the frame (7), respectively. Then the adsorbent layer (4) was prewetted with the mobile phase solution (Fig. 2a). In the subsequent step, the cover (10) of the PPEC chamber was pressed against the adsorbent layer using an hydraulic press (13), and the six lines of the on-line sample application/injection device, each composed of tubing (22), a reservoir (21) and syringe (23), was filled up with a solution of the same composition as in the mobile phase (Fig. 4). The syringes (23) were combined with a stepping motor (17 in Fig. 3), which moved the syringe pistons to suck the solution from the reservoirs (21) through the tubing (22). When the potential was applied to the electrodes, six sample solutions, each of 25 μL volume, were injected into the tubing with six microsyringes (20) (Figs. 3, 4). During this operation, zones of injected sample solutions migrated through grooves in the adsorbent layer in the perpendicular direction to the direction of electroosmotic flow of the mobile phase. Under such conditions, some portions of the sample solutions flowing through each groove were introduced by this electroosmotic flow into the separation system. The rest of the sample solution, which did not enter the separation system, was transferred from the grooves to the syringes (23) through the tubing combined with them (Fig. 4). After the separation process, a high voltage supply was switched off, the mobile phase solution was sucked out of the electrode compartments, and the chromatographic plate was removed from the PPEC chamber for typical evaluation.

## Results and Discussion

### Progress in Construction of the Apparatus for PPEC

As mentioned above, regarding the operation of the device equipped with two partitions previously proposed by our group [14], the mobile phase prewetting solution had to be removed from the PPEC chamber to enable the cover pressing on an adsorbent layer. In the modified device (with one partition, Fig. 2a, b), the stage of removal of the mobile phase solution from the PPEC chamber after the prewetting process with a sample spot under the partition was eliminated. The elimination of this step is very important for further application of the PPEC mode in routine analysis for several reasons. First of all, it considerably facilitates prewetting the adsorbent layer before running the PPEC process. Problems concerned with drops of the mobile phase solution being left on the adsorbent layer after the prewetting process and evaporation of the solvent from the prewetted adsorbent layer are completely eliminated. The proposed modification of the equipment for PPEC enables performing the separation process without the risk of adsorbent layer damage because the stage of removing the mobile phase solution was eliminated. In this way, the number of manual operations, which are difficult for validation, is reduced.

However, applications of sample solutions and the PPEC separation process have to be performed individually and independently of the separation process with the equipment presented in Fig. 2. Under such conditions, both stages (the sample application and separation process) seem to be difficult to automate. Moreover, application of samples using an aerosol applicator is time consuming (in the case of quantitative analysis, it requires even up to 1 h depending on the sample number, application volume and solvent type).

Further modification of equipment for PPEC was dictated by the necessity for shortening the most time-consuming stage sample application. A new mode of on-line sample application with the prototype device (Figs. 3, 4) enables simultaneous application/injection of six samples on the chromatographic plate in a stream of the mobile phase. The main advantages of on-line sample application in PPEC are the considerable shortening of the separation procedure and running of the PPEC process under the equilibrated conditions of the mobile-stationary phase system. An additional advantage of this approach is eliminating the risk of excessive dispersion of the starting substance zones during the prewetting of an adsorbent layer of the chromatographic plate.

During PPEC experiments, Joule heat is generated, leading to a temperature increase in the separating system. Cooling the PPEC chamber using circulating liquid can reduce this effect [3]. Our device was equipped with a cooling/heating plate (24), which also enables inserting and removing the chromatographic plate in and out of the PPEC chamber, respectively (see Figs. 3, 4). Temperature control of the electrochromatographic system between 19.5 and 20.5 °C provided stable, repeatable conditions during separation.

### Separation of Solutes

**Fig. 5:**
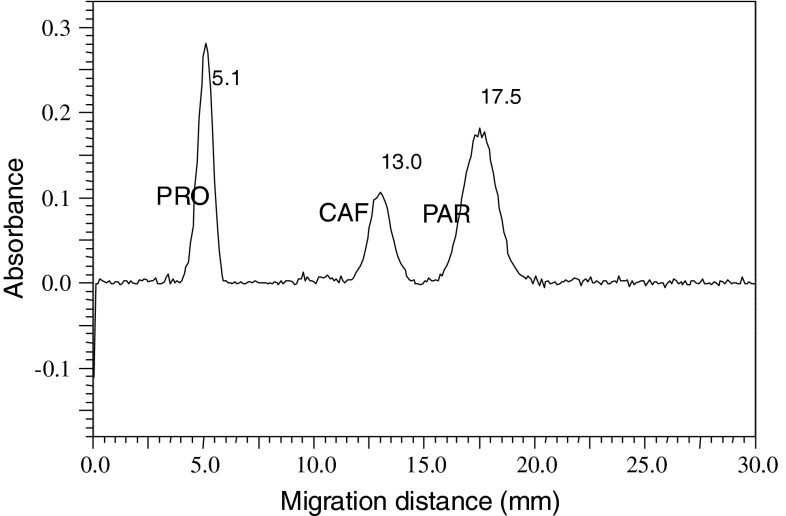
Electrochromatogram of the sample mixture of propyphenazone (*PRO*), caffeine (*CAF*) and acetaminophen (*PAR*) on the RP 18 W HPTLC chromatograpic plate (Merck) with 22.5 % acetonitrile in pH 5.0 buffer (1 mM citric acid, 2 mM disodium hydrogen phosphate), polarization potential 1.0 kV, development time 2 min

**Fig. 6:**
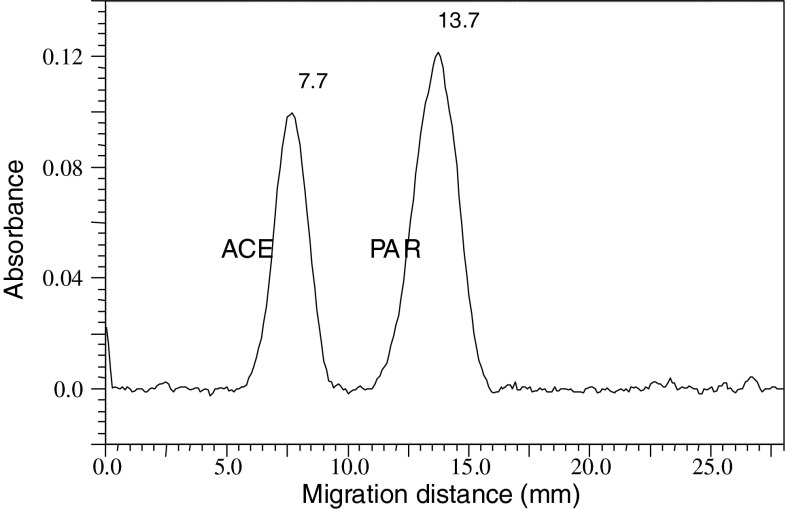
Electrochromatogram of the sample mixture of acetanilide (*ACE*) and acetaminophen (*PAR*) on RP18 W (LiChrospher, Merck) chromatographic plates with 20 % acetonitrile in pH 5.0 buffer (0.5 mM citric acid, 1 mM disodium hydrogen phosphate), polarization potential 1.0 kV, development time 3 min

Optimization of separation conditions of the components of the cefalgin preparation (propyphenazone, caffeine and acetaminophen) was performed based on our previously presented results [13]. For separation of substance zones of acetaminophen and acetanilide (internal standard), the electrochromatographic system was slightly modified. Typical electrochromatograms showing the separation of the components of the cefalgin preparation and separation of acetaminophen and acetanilide are presented in Figs. 5 and 6, respectively. In Table 1, parameters that characterize the separation quality of the sample mixtures with the PPEC technique are presented. As can be seen, all peaks are well shaped and symmetrical (tailing factor, *T*_F_, ranging from 0.92 to 1.02, and peak asymmetry, *A*_S_, between 0.9 and 1.2). It is true that the separated mixture was simple; however, the electrochromatographic system enables good selectivity and resolution that is really good (*R*_S_ > 3.18). Performance was also satisfactory in the preliminary study. Values of plate height, *H*, are between 29 and 59 μm. In our opinion, the obtained peak dispersion is concerned with features of the prototype of the on-line sample application/injection device, which was probably not running under fully optimum conditions. We suppose that optimization of the sample volume, reduction of the dead volume of the grooves in the adsorbent layer, standardized production of the grooves and of the partition of PPEC chamber with tubing in it will improve the separation efficiency.

**Table 1:** Values of parameters characterizing the PPEC systems obtained with aerosol sample application and on-line sample application/injection

Parameters	PPEC with aerosol mode of sample application			PPEC with on-line application/injection	
	PRO	CAF	PAR	ACE	PAR
*z* (mm)	5.1	13.0	17.5	7.7	13.7
*R*_s_	8.4	3.2		3.3	
*T*_F_	0.95	0.97	1.02	0.92	1.02
*A*_s_	0.90	1.2	1.0	0.98	1.0
*H* (μm)	39.9	29.1	57.5	48	59

*PRO* propyphenazone, *CAF* caffeine, *PAR* acetaminophen, *z* migration distance of solute zones, *R*_S_ resolution, *T*_F_ tailing factor at *W*_0.05,_ *A*_S_ peak asymmetry at *W*_0.1_, *H* height of theoretical plate

### Calibration Curves

The data (presented in Table 2) show that both methods (PPEC with aerosol sample application and PPEC with on-line sample application) are characterized by high correlation coefficient values, *r*, of the relationship peak area versus sample quantity (*r* > 0.99). However, the data obtained with the former method show the best fitting to polynomial regression equations contrary to those when the latter was used; then the experimental data show the best fit to the linear regression equation.

**Table 2:** Parameters of calibration curves obtained with PPEC with aerosol sample application and with on-line sample application/injection

PPEC technique	Substance	Equation	*R* ^2^	Correlation coefficient (*r*)
Aerosol sample application	PRO	*Y* = −0.0033*x* ^2^ + 0.1329*x* + 0.1048	0.9974	0.9987
	CAF	*Y* = −0.0121*x* ^2^ + 0.2312*x* + 0.0062	0.9970	0.9985
	PAR	*Y* = −0.0015*x* ^2^ + 0.1238*x* + 0.3547	0.9961	0.9980
On-line sample application/injection	PAR	*Y* = 0.9328*x* + 0.111	0.9905	0.9952

### Precision and Accuracy

Comparison of the precision of PPEC with aerosol sample application and with on-line application/injection was performed based on *RSD* values of the peak areas of the solutes. Additionally, *RSD* values of quantitative determination of the active substances were compared. Obtained results are presented in Tables 3 and 4. It is evident that the dispersion of the results for PPEC with on-line sample application (*RSD* equal to 1.54 %) is about two times lower than that for PPEC with the aerosol sample application mode (*RSD* values ranged from 3.10 to 3.84 %). The results are similar to those for determination of the content of active substances in pharmaceutical preparation. The *RSD* value for the former mode is equal to 1.56 %; for the latter mode it ranged from 4.43 to 5.87 %. The higher reproducibility of the peak area when PPEC was used with on-line application/injection can be explained as follows. If substance zones are applied/injected on-line into the stream of the mobile phase after the prewetting process, then separation was conducted in the equilibrated conditions of the mobile-stationary phase system from the very beginning stage. Moreover, this mode of sample application eliminates any additional distortion of the starting substance zones that takes place during the pre-wetting process of the adsorbent layer with the mobile phase solution. An increase in the repeatability of the on-line sample application mode could also be partially caused by using the cooling plate, which maintains a constant temperature during the experiments. Nurok et al. showed that the separation efficiency was sensitive to the temperature of the PPEC separation system [3]. It should be emphasized that there is no possibility to obtain reproducible sample volumes for each track/path on the chromatographic plate with the on-line application/injection mode presented in this article. Therefore, the internal standard addition method was applied to overcome this difficulty. Comparison of the recovery values, which characterize the accuracy of the PPEC technique with two sample application methods, is shown in Table 5. The results confirm the above discussions. It can be seen that better results were obtained when PPEC with on-line sample application/injection was used (the recovery ranged from 97.80 to 103.10 %, while this value for PPEC with aerosol sample application for the same substance ranged from 101.14 to 103.10 % and from 95.60 to 107.36 % for other substances).

**Table 3:** Comparison of precision of PPEC obtained with aerosol sample application and with on-line sample application/injection (six independent samples)

PPEC technique with	Substance	Intraday			Interday		
		x	SD	RSD (%)	x	SD	RSD (%)
Aerosol sample application	PRO	1.0118	0.0388	3.84	1.0225	0.0410	4.05
	CAF	0.5732	0.0212	3.70	0.5631	0.0365	6.48
	PAR	1.8611	0.0576	3.10	1.8249	0.1139	6.24
On-line sample application/injection	PAR	0.9392	0.0145	1.54	0.9335	0.0321	3.44

*x* the average of the peak area,* SD* standard deviation, *RSD* relative standard deviation

**Table 4:** Results of quantitation of active substances in the tablets: cefalgin and paracetamol with PPEC with aerosol sample application and PPEC with on-line sample application/injection, respectively

PPEC technique with	Declared content (mg)	Found (mg)	% of declared content	RSD (%)
Aerosol sample application	PRO (150)	152.90	101.93	5.87
	CAF (50)	50.69	101.38	4.43
	PAR (250)	260.06	104.02	4.91
On-line sample application/injection	PAR (500)	497.17	99.43	1.56

**Table 5:** Results of determination of the accuracy of PPEC with aerosol sample application and with on-line sample application/injection

PPEC technique with	Substance	In tablet mass (mg)	Standard addition (mg)	Found (mg)	Recovery (%)
Aerosol sample application	PRO	76.50	55.82	133.83	101.14
			71.72	150.24	101.36
			89.82	170.55	102.54
	CAF	25.36	20.69	49.44	107.36
			24.90	50.45	100.38
			28.93	51.90	95.60
	PAR	130.19	96.53	239.46	105.62
			125.53	258.63	101.14
			152.35	287.33	101.70
On-line sample application/injection	PAR	260.50	125.41	397.89	103.10
		261.02	247.51	497.36	97.80
		258.44	353.12	604.00	98.76

### Effectiveness

A considerable advantage of PPEC with on-line sample application is that both stages of the PPEC process, the sample application and separation of sample components, are performed in individual procedures, which is different from PPEC with sample application using an aerosol applicator. Under the latter conditions, both stages (sample application and separation process) seem to be difficult to automate.

The time necessary for all steps of the separation process (from sample application to final chromatogram registration) using the previously reported chromatographic techniques (TLC [13, 15], PPEC with the chamber adapted for separation with 2-cm-wide plates [13] and PPEC with two chamber partitions adapted for separation with 10-cm-wide plates [14]) and investigated PPEC techniques with chambers using aerosol sample application and on-line sample application/injection (both adapted for electrochromatogram development with 10-cm-wide plates) is compared in Table 6. The comparison was made for the six samples because the prototype of the device for on-line sample application enables simultaneous determination of six samples on one chromatographic plate. The number of applied zones of solutes on the chromatographic plate depends on the length of the applied bands, and in this case depends on the distance between the inlet and outlet Teflon tubing in the partition (12) (Fig. 4). Replacement of the partition with another one enables changing the above-mentioned application/injection parameters. PPEC equipped with on-line sample application/injection proved to be the most efficient. Determination of the six samples can be performed within 14 min. The time is almost four times shorter in comparison to that of TLC. PPEC with on-line application/injection enables separating many samples in a short time because of elimination of the time-consuming stage of sample application using an aerosol applicator. Separation time with the PPEC technique can be even considerably shorter than described in this article because of the application of higher polarization voltage [13]. In Table 6, the time of aerosol application of one sample is 5 min. In practice, that value varies within a large range and depends on the vapor pressure of the solvent in which the sample is dissolved (in this case methanol) and the volume applied. The manufacturer recommends application rates from 50 nLs^−1^ for water to 250 nLs^−1^ for acetone (for methanol 150 nLs^−1^). The aerosol application method becomes a time-consuming stage for an analysis of multiple samples. As apparent from Table 6, the application of six spots lasts up to 30 min, while for on-line application this is only 1 min.

**Table 6:** The analysis times (min) of six samples using different techniques (details in the text)

Operation	TLC [13, 15]	PPEC [13]	PPEC [14]	PPEC^a^	PPEC^b^
Application	30	30	30	30	1
Equlibration	15	6	1	1	1
Separation process	5	12	2	2	3
Detecion	6	6	6	6	6
Additional operations	1	36	3	2	3
Total	57	90	42	41	14

^a^PPEC with aerosol mode of sample application

^b^PPEC with on-line sample application/injection

## Conclusions

Preliminary results show that PPEC with on-line application/injection enables performing the separation of many samples in a considerably shorter time than with off-line sample application. In addition, the separation process can be performed under equilibration conditions similar to those for column liquid chromatography techniques. In PPEC, on-line sample application/injection leads to elimination of any additional starting solute zone distortion in comparison to aerosol application. However, the proposed mode of on-line sample application/injection in the PPEC system requires an internal standard in a sample if this technique is to be used for quantitative analysis. The developed prototype device still needs further investigations, e.g. construction of a PPEC chamber for wider plates that enables separating a larger number of samples simultaneously. Our results demonstrate progress in the range of PPEC development and are promising for automation of this technique in the future, which has not been achieved so far for thin-layer chromatography.
